# ANXA1: An Important Independent Prognostic Factor and Molecular Target in Glioma

**DOI:** 10.3389/fgene.2022.851505

**Published:** 2022-05-31

**Authors:** Dongdong Zhang, Wenyan Wang, Huandi Zhou, Linlin Su, Xuetao Han, Xinyuan Zhang, Wei Han, Yu Wang, Xiaoying Xue

**Affiliations:** ^1^ Department of Radiotherapy, The Second Hospital of Hebei Medical University, Shijiazhuang, China; ^2^ Department of Central Laboratory, The Second Hospital of Hebei Medical University, Shijiazhuang, China; ^3^ Department of Oncology, The First Hospital of Qinhuangdao, Qinhuangdao, China; ^4^ Department of Oncology, Hebei General Hospital, Shijiazhuang, China

**Keywords:** ANXA1, glioma, prognosis, ECM, focal adhesion, molecular target

## Abstract

**Objective:** The expression, prognosis, and related mechanisms of ANXA1 are investigated in glioma, with the objective to find potential therapeutic molecular targets for glioma.

**Methods:** We analyzed the gene expression of ANXA1 using glioma-related databases, including the Chinese Glioma Genome Atlas (CGGA) database, The Cancer Genome Atlas (TCGA) database, and the Gene Expression Omnibus (GEO) database. Moreover, we collected the sample tissues and corresponding paracancerous tissues of 23 glioma patients and then conducted a Western blot experiment to verify the expression and correlate survival of ANXA1. Moreover, we generated survival ROC curves, performing univariate and multivariate Cox analyses and the construction of the nomogram. Differential expression analysis was conducted by high and low grouping based on the median of the ANXA1 gene expression values. We conducted Kyoto Encyclopedia of Genes and Genomes (KEGG) enrichment analysis and Gene Set Enrichment Analysis (GSEA) to explore possible mechanisms, and gene co-expression analysis was also performed.

**Results:** The results showed that the ANXA1 expression level was higher in gliomas than in normal tissues, and a high expression level of ANXA1 in gliomas was associated with poorer prognosis. The independent prognosis analysis showed that the ANXA1 gene was an independent prognostic factor of glioma. In the analysis of KEGG and Gene Set Enrichment Analysis (GSEA), it is shown that ANXA1 may play an important role in glioma patients by affecting extracellular matrix (ECM)–receptor interaction and the focal adhesion signal pathway. The core genes, including COL1A1, COL1A2, FN1, ITGA1, and ITGB1, were screened for gene correlation and prognosis analysis. The expression level of the five genes was verified by qPCR in glioma. We concluded that these five core genes and ANXA1 could play a synergistic role in gliomas.

**Conclusion:** The results indicated that a high expression level of ANXA1 leads to worse prognosis and ANXA1 is an independent prognostic factor and a potentially important target for the treatment of gliomas.

## Introduction

Glioma is an intracranial tumor originating from glial cells, and it is the most common intracranial primary tumor, accounting for approximately 80% of intracranial malignant tumors (Ostrom et al., 2014). Glioma is often accompanied by local invasion, especially glioblastoma (GBM), which invades and destroys the surrounding normal brain tissue, and the degree of malignancy is high with a poor prognosis (Weller et al., 2015). High-grade gliomas (HGGs) are the most common primary brain malignancies, predicting a 5-year survival rate of less than 5% (Ostrom et al., 2014). These tumors may arise *de novo* as isocitrate dehydrogenase (IDH)–wildtype GBMs or develop from progressive lower-grade gliomas (LGGs; defined as World Health Organization (WHO) grades 2–3) (Aldape et al., 2015). According to the WHO, gliomas were divided into grades I–IV by pathological characteristics; grade II–IV gliomas are the largest entity in the group of intracranial brain tumors, with a survival rate of more than 10 years (grade II) to less than 1 year (grade IV) (Cordier et al., 2016). However, the therapeutic effect is limited with conventional treatment of gliomas, including complete surgical resection and postoperative radiotherapy and chemotherapy (Stupp et al., 2005; Bush et al., 2017). In recent years, molecular-targeted therapy and immunotherapy have achieved good results in many tumors, such as colorectal cancer, breast cancer, and lung cancer (Naylor et al., 2016; Esteva et al., 2019; Ganesh et al., 2019; Piawah and Venook, 2019). However, due to the existence of the blood–brain barrier in the brain and the unique tumor microenvironment in glioma, the therapeutic effect is poor (Quail and Joyce, 2017). However, at present, molecular-targeted therapy and immunotherapy have achieved some effective results. For example, vemurafenib has demonstrated long-lasting antitumor activity in some patients with *BRAFV600E* mutant glioma (Kaley et al., 2018). Neoadjuvant administration of PD-1 blockers enhances local and systemic antitumor immune responses (Cloughesy et al., 2019). With the advent of the molecular era, the classification of gliomas has changed. Combined with pathological and molecular characteristics, the classification of gliomas by the WHO in 2016 included molecular markers, such as IDH mutation and 1p19q (Louis et al., 2016). In 2021, the latest central nervous system tumor guidelines of the fifth edition of the WHO confirmed the importance of molecular typing. In the grading of gliomas, the importance of grading according to molecular characteristics is more prominent. For example, loss of CDKN2A/B homozygosity is closely related to poor prognosis, especially in WHO grade III glioma with loss of CDKN2A/B homozygosity, where the prognosis is similar to WHO grade IV glioma. Therefore, CDKN2A/B has been included in the molecular diagnosis of gliomas (Louis et al., 2021). However, molecular typing still needs to be further explored to identify more molecular targets for better diagnosis and treatment of clinical patients. Therefore, we explore new targets of glioma to provide new therapeutic targets.

Annexin is a well-known calcium-regulated and phospholipid-dependent membrane-binding protein (Lim and Pervaiz, 2007). ANXA1 is a member of the annexin family, which is involved in many important biological processes, such as inflammation, phagocytosis, proliferation, differentiation, and apoptosis (Monastyrskaya et al., 2009; Xia et al., 2020). Many studies have shown that ANXA1 is associated with the occurrence, invasion, and metastasis of cancer (Mussunoor and Murray, 2008; Guo et al., 2013). ANXA1 has been extensively studied in gastric cancer and breast cancer (Maschler et al., 2010; Cheng et al., 2012). Some studies in gliomas have shown that ANXA1 is a risk prognostic factor (Xu et al., 2017; Luo et al., 2021; Wei et al., 2021) Therefore, we investigated the expression, prognosis, and related mechanisms of ANXA1 using a public database to provide new markers and potential therapeutic targets for glioma patients.

## Materials and Methods

### Data Acquisition and Download

The GSE4290, GSE7696, GSE29796, and GSE50161 expression sequences and clinical data were downloaded from the Gene Expression Omnibus (GEO, https://www.ncbi.nlm.nih.gov/geo/) database for gene expression analysis. The GSE4290 dataset included 23 normal samples, which were obtained from epilepsy patients and used as nontumor samples, and 153 tumor samples, which included 26 astrocytomas, 50 oligodendrogliomas, and 77 glioblastomas (Sun et al., 2006). The GSE7696 dataset included 80 glioblastoma specimens and four nontumor brain samples (Murat et al., 2008). The GSE29796 dataset included 20 normal samples and 52 tumor samples, and these samples were based on matching patients with disease and/or pathology, including 20 cases of epileptic pathology and 52 cases of glioma (Auvergne et al., 2013). The GSE50161 dataset collected samples from surgical brain tumors and normal brains, including 13 normal samples and 117 tumor samples (Griesinger et al., 2013). In addition, the expression sequences and clinical data of 1,018 samples were downloaded from the Chinese Glioma Genome Atlas (CGGA, http://www.cgga.org.cn/) database, and the expression sequences and clinical data of 592 samples were downloaded from The Cancer Genome Atlas (TCGA, https://portal.gdc.cancer.gov/) database (Jiang et al., 2016), which included 449 LGG samples and 143 glioblastoma (GBM) samples. Before further analysis, we performed a log2 transformation on RNA-sequencing data. All sample databases were screened to remove samples that had omitted clinical information.

### Acquisition of 23 Patient Tissues and Collection of Clinical Information

We collected sample tissues and corresponding paracancerous tissues (distance from tumor edge >2 cm) from 23 glioma patients, including eight GBM samples and their corresponding paracancerous tissues and 15 LGG samples and their corresponding paracancerous tissues. Under the guidance of neurosurgery experts, gliomas and corresponding paracancerous tissues were obtained from surgery. All samples were obtained with the informed consent of the patients and their families, and the present study was approved by the Ethics Committee of the Second Hospital of Hebei Medical University. We followed up 23 glioma patients from January 2015 to December 2021, and we collected the following information: patient age, gender, grade, radiotherapy chemotherapy, and IDH mutation.

### Expression Analysis of the ANXA1 Gene

The GSE4290, GSE7696, GSE29796, and GSE50161 datasets were downloaded from the GEO database. The expression values of ANXA1 in glioma and normal brain tissues were imported into GraphPad Prism 8 software for analysis followed by verification with Gene Expression Profiling Interactive Analysis (GEPIA, http://gepia.cancer-pku.cn/) and Human Protein Atlas online analysis (https://www.proteinatlas.org/). Moreover, Western blot analysis was also performed to verify the expression level of ANXA1.

### Analysis of Clinical Prognosis and Clinicopathological Features of ANXA1

The clinical prognosis of ANXA1 was analyzed based on the transcriptome data and clinical information of 1,018 cases from the CGGA database. The expression level of ANXA1 was divided into high and low groups according to the median value. The survival curves of different expression levels of ANXA1 were drawn using the “survival” and “survivminer” software packages in R version 4.0.5. We used the “survival ROC” software package and the Kaplan–Meier method to calculate the receiver operator characteristic (ROC) curve of ANXA1 at 1, 3, and 5 years. The prognostic value of ANXA1 was further evaluated by univariate and multivariate Cox regression analyses, with a significance level of *p* < 0.001. To verify the accuracy of the ANXA1 prognosis, TCGA data were used to generate the survival and ROC curves and to perform univariate and multivariate analyses. Finally, based on the CGGA data, we used the “survival” and “RMS” software packages in R version 4.0.5 to construct a nomogram for 1-year, 2-year, and 3-year survival using the clinicopathological characteristics of ANXA1 expression. Subsequently, calibration curves were drawn to assess the accuracy of matching between predicted survival and actual survival. In addition, the correlation between the expression of ANXA1 and clinicopathological features was analyzed using the “beeswarm’’ package in R version 3.6.3.

### Analysis of Differentially Expressed Genes and Signal Pathway Mechanism

The mRNA sequencing data of the glioma database were normalized using the CGGA. The median expression value of ANXA1 was divided into high and low groups for differential expression analysis, and the Differentially Expressed Genes (DEGs), including significantly upregulated and downregulated genes, were screened by adjusted *p* < 0.05 and absolute log2 fold change (FC) > 1. The “limma” and “ggplot2” software packages (Ritchie et al., 2015) were used to generate a volcano plot to visualize the DEGs, and the 30 genes with the most significant upregulation and downregulation were selected to generate a heat map of the DEGs using the “pheatmap” software package in R version 4.0.5. Finally, mechanism analysis of the DEGs was performed using Metascape (https://metascape.org/). In addition, GSEA (https://www.gsea-msigdb.org/) was also utilized to indirectly explain the potential mechanism of ANXA1 function. When NES > 1, *p* < 0.05, and FDR < 0.05, the gene set was considered the enrichment group.

### Construction of the Protein–Protein Interaction Network of Differentially Expressed Genes

The median expression value of ANXA1 was divided into high and low groups using the cutoff of adjusted *p* < 0.05 and absolute log2 fold change (FC) > 1 to screen the DEGs, and a high threshold (0.7) binding degree was set for node screening through the STRING (https://string-db.org/) analysis to filter out the unconnected proteins. The remaining 1,034 proteins were imported into Cytoscape visualization software (version: 3.7.2). After installing the cytoHubba plug-in, the top 50 hub genes were screened by degree topology analysis.

### Coexpression Analysis of ANXA1 and Core Genes

We obtained the core genes of ECM–receptor interaction and focal adhesion pathways by GSEA. Through a protein–protein interaction (PPI) network analysis, the DEGs were imported into Cytoscape visualization software to screen the top 50 hub genes by using degree topology analysis. Five core genes were obtained through the intersection of the Venn diagram for gene correlation analysis and coexpression analysis. The relative expression levels of the five genes in glioma and their corresponding paracancerous tissues were further analyzed by qPCR.

### Chemical Reagents and Antibodies

The RIPA lysate (product number: p00138b) was purchased from Beyotime (Shanghai, China). Protein phosphatase inhibitors and BCA protein concentration determination kits were purchased from Solarbio (Beijing, China). ANXA1 antibody (catalog number: 21990-1-AP) and β-tubulin antibody (catalog number: 10094-1-ap) were obtained from Proteintech (Chicago, IL, United States). The anti-rabbit IgG HRP-conjugated antibody was purchased from Cell Signaling Technology. An ECL chromogenic solution (product number: BL520A) was obtained from Biosharp.

### Tissue Protein Extraction and Western Blot

Tissue samples were removed from the −80°C freezer and thawed on ice. An RIPA lysis buffer, containing protein phosphatase inhibitor, was added to each sample, and the samples were homogenized using a homogenizer. The samples were centrifuged at 4°C and 12,000 rpm for 10 min, and the supernatants were collected. The protein concentrations were determined using a commercially available BCA protein concentration kit. After denaturation, 10–20 µg of the protein was separated with 10% SDS-PAGE and then transferred onto PVDF membranes (Merck Millipore Ltd.). The membranes were then blocked with 5% nonfat milk powder for 1 h and incubated with primary antibodies (ANXA1 antibody, 1:2000; and β-tubulin antibody, 1:1000) at 4°C overnight. The membranes were then washed thrice with TBST (10 min each wash) followed by incubation with the appropriate secondary antibody (anti-rabbit IgG, 1:2000). The membranes were then washed thrice with TBST (10 min each wash) followed by development and imaging using a chemiluminescence photodocumentation system.

### RNA Isolation and qPCR

Total RNA was extracted from the remaining eight glioma samples and corresponding paracancerous tissues with TRIzol reagent (Invitrogen). The quality of the total RNA of these samples was evaluated by Nanodrop 2000c and agarose gel electrophoresis. Complementary DNA (cDNA) was synthesized using a revert aid first-strand cDNA synthesis kit (Thermo Fisher Scientific, Waltham, MA, United States). Quantitative PCR was performed with SYBR Green (Hieff qPCR SYBR Green Master Mix, YEASEN, Shanghai, China) on a qPCR system (Model No. CFX96^TM^ Option Module, Bio-Rad, United States). The specific primers of each gene were used to analyze the expression of these extracted tissue samples. All samples were standardized according to the expression of the gene-encoding human glyceraldehyde 3-phosphate dehydrogenase (GAPDH) as a reference. Relative expression levels were calculated as 2^−[(Ct of target gene)−(Ct of GAPDH)]^. The sequences of the primers are listed in Supplementary Table S1.

### Statistical Analysis

Based on public databases, the following software programs and online tools were used for analysis: GraphPad Prism 8 software was used for gene expression analysis; R software (version: 4.0.1, http://www.r-project.org/) was used for survival prognosis analysis and gene correlation analysis; R software (version: 3.6.3, http://www.r-project.org/) was used for clinicopathological characteristics analysis; Metascape online website and GSEA software were used for mechanism analysis; and the GEPIA online website was used for core gene prognosis analysis. When the results met the requirements of *p* < 0.05 and FDR < 0.05, they were considered statistically significant. In addition, we used Western blot analysis to verify the expression levels of 23 gliomas and their corresponding paracancerous tissues. The protein gray values were measured by ImageJ software, and the results were imported into GraphPad Prism 8 software. qPCR was used to verify the expression of the five core genes in the remaining eight pairs of samples. *p* < 0.05 was considered statistically significant.

## Results

### Article Workflow and Sample Information

**FIGURE 1:**
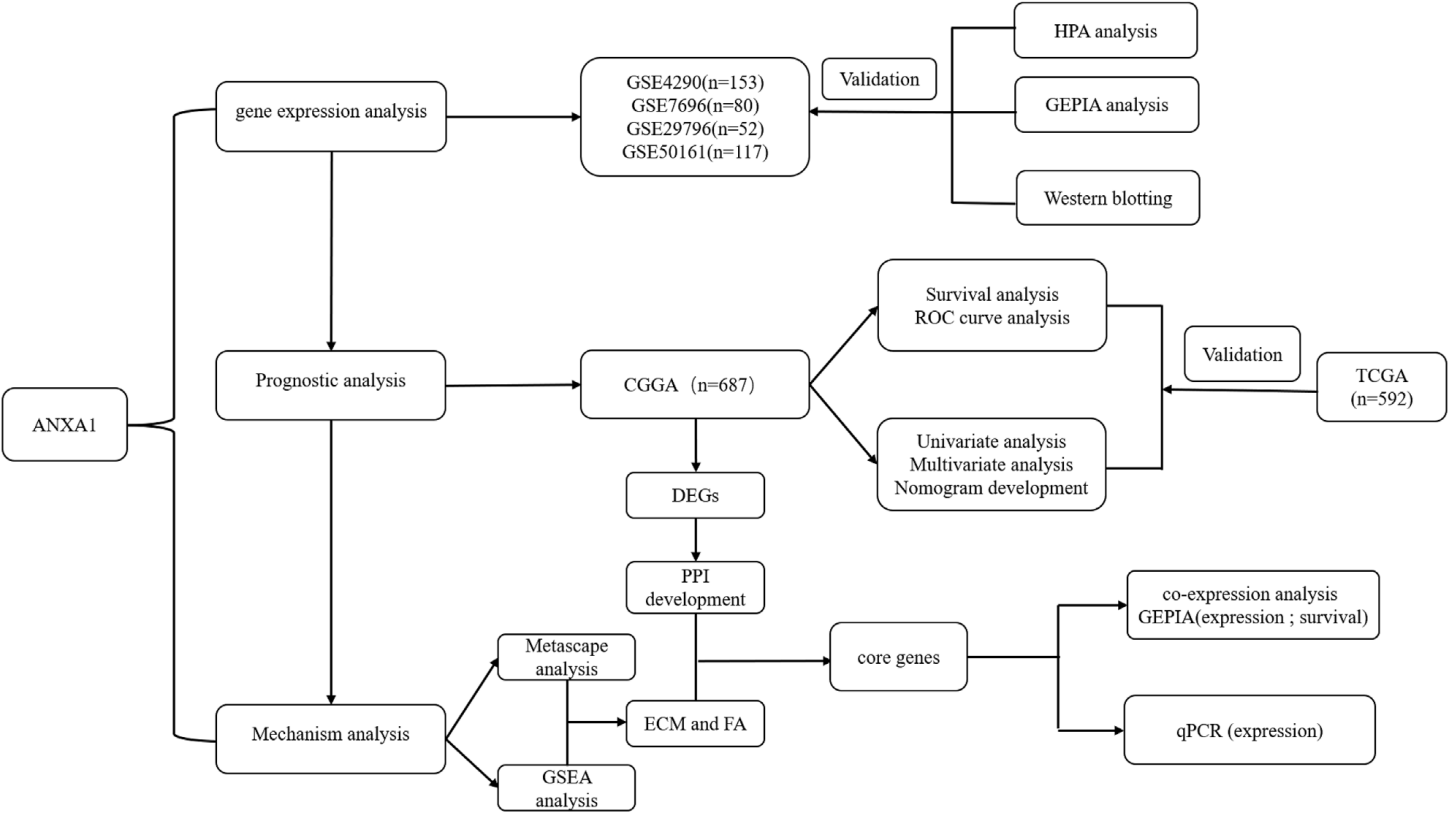
Working diagram of the study. Abbreviations: GEO: Gene Expression Omnibus; CGGA: Chinese Glioma Genome Atlas; DEGs: differentially expressed genes; GSEA: Gene Set Enrichment Analysis; PPI: protein–protein interaction; ECM: extracellular matrix; FA: focal adhesion; HPA: Human Protein Atlas; GEPIA: Gene Expression Profiling Interactive Analysis; TCGA: The Cancer Genome Atlas.

The workflow of the present study is shown in Figure 1. We downloaded the glioma sample information of the GSE4290, GSE7696, GSE29796, and GSE50161 datasets from the GEO database with 153, 80, 52, and 117 glioma samples and 23, 4, 20, and 52 normal samples, respectively. The clinicopathological information, including age, grade, category, 1p19q deletion status, and IDH mutation status, was obtained from the CGGA and TCGA databases. However, the TCGA database lacked clinical chemoradiotherapy and MGMT methylation data, which were only obtained from the CGGA database. The clinical information and categorical data of glioma patients from the CCGA and TGGA databases are shown in Table 1. The clinical information for the 23 glioma patients who provided samples for Western blot analysis is shown in Supplementary Table S2.

**TABLE 1:** Clinical information materials of gliomas. CGGA: 686 glioma samples; TCGA: 592 glioma samples; clinical features: grade, gender, age, IDH mutation, 1p19q codeletion, MGMT, radiotherapy, and chemistry.

	CGGA (*n* = 686)		TCGA (*n* = 592)	
	Case	Proportion (%)	Case	Proportion (%)
WHO grade				
II	177	25.8	211	35.6
III	226	32.9	238	40.2
IV	283	41.3	143	24.2
Gender				
Male	399	58.2	344	58.1
Female	287	41.8	248	41.9
Age				
≥42	379	55.2	349	59.0
<42	307	44.8	243	41.0
IDH mutation				
Yes	371	54.1	372	62.8
No	315	45.9	220	37.2
1p19q codeletion				
Yes	141	20.6	149	25.2
No	545	79.4	443	74.8
MGMT methylation				
Yes	386	56.3	—	—
No	300	43.7	—	—
Radiotherapy				
Yes	544	79.3	—	—
No	142	20.7	—	—
Chemotherapy				
Yes	501	73.0	—	—
No	185	27.0	—	—

### ANXA1 is Overexpressed in Glioma

**FIGURE 2:**
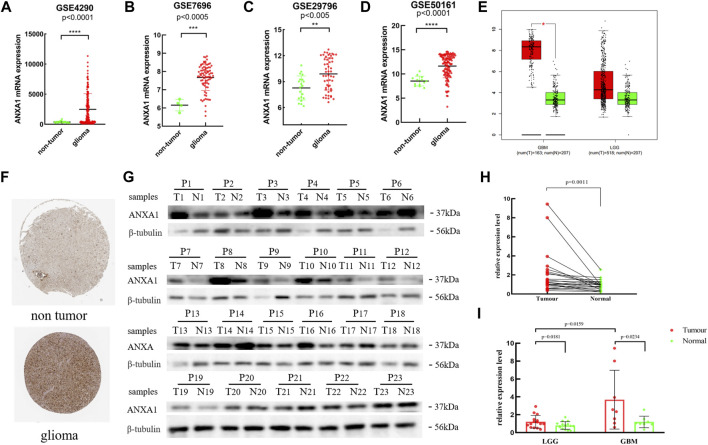
Expression level of the ANXA1 gene in glioma and normal tissues. **(A)** GSE4290 data set. **(B)** GSE7696 data set. **(C)** GSE29796 data set. **(D)** GSE50161 data set. **(E)** Based on the GEPIA online website, the expression level of ANXA1 in glioma and normal samples. **(F)** Based on The Human Protein Atlas online website, the expression level of ANXA1 in the brain tissues of glioma (Intensity: strong; Quantity: 75%–25%) and normal controls (Intensity: weak; Quantity: 75%–25%). **(G)** Western blotting images of 23 glioma tissues and their corresponding paracancerous samples (P1–P8: GBM; P9–P23: LGGs). **(H)** Paired-sample *t*-test of 23 glioma tissues and their corresponding paracancerous tissues. **(I)** Paired-sample *t*-test of GBM and LGG tumor tissues and their corresponding paracancerous tissues, and independent-sample *t*-test of eight GBM and 15 LGG samples.

Based on the expression data for gliomas and normal tissues in the GSE4290, GSE7696, GSE29796, and GSE50161 datasets, the ANXA1 gene was significantly highly expressed in gliomas (Figures 2A–D), and the results were verified by GEPIA and Human Protein Atlas online analyses (Figures 2E,F). In addition, we evaluated 23 glioma sample tissues and their corresponding paracancerous tissues by Western blot analysis (Figure 2G), and the results showed that the ANXA1 expression levels were significantly higher in the 23 glioma sample tissues than their corresponding paracancerous tissues (*p* < 0.05, Figure 2H). A paired-sample *t*-test was performed separately in 15 LGG samples and their corresponding paracancerous tissues and in eight GBM samples and their corresponding paracancerous tissues. In addition, an independent-sample *t*-test was also performed in eight GBM samples and 15 LGG samples. The results demonstrated that ANXA1 was overexpressed in gliomas with higher expression in GBM samples than in LGG samples (*p* < 0.05, Figure 2I), supporting that ANXA1 is correlated with a higher grade of gliomas.

### High Expression of ANXA1 has a Poor Prognosis

**FIGURE 3:**
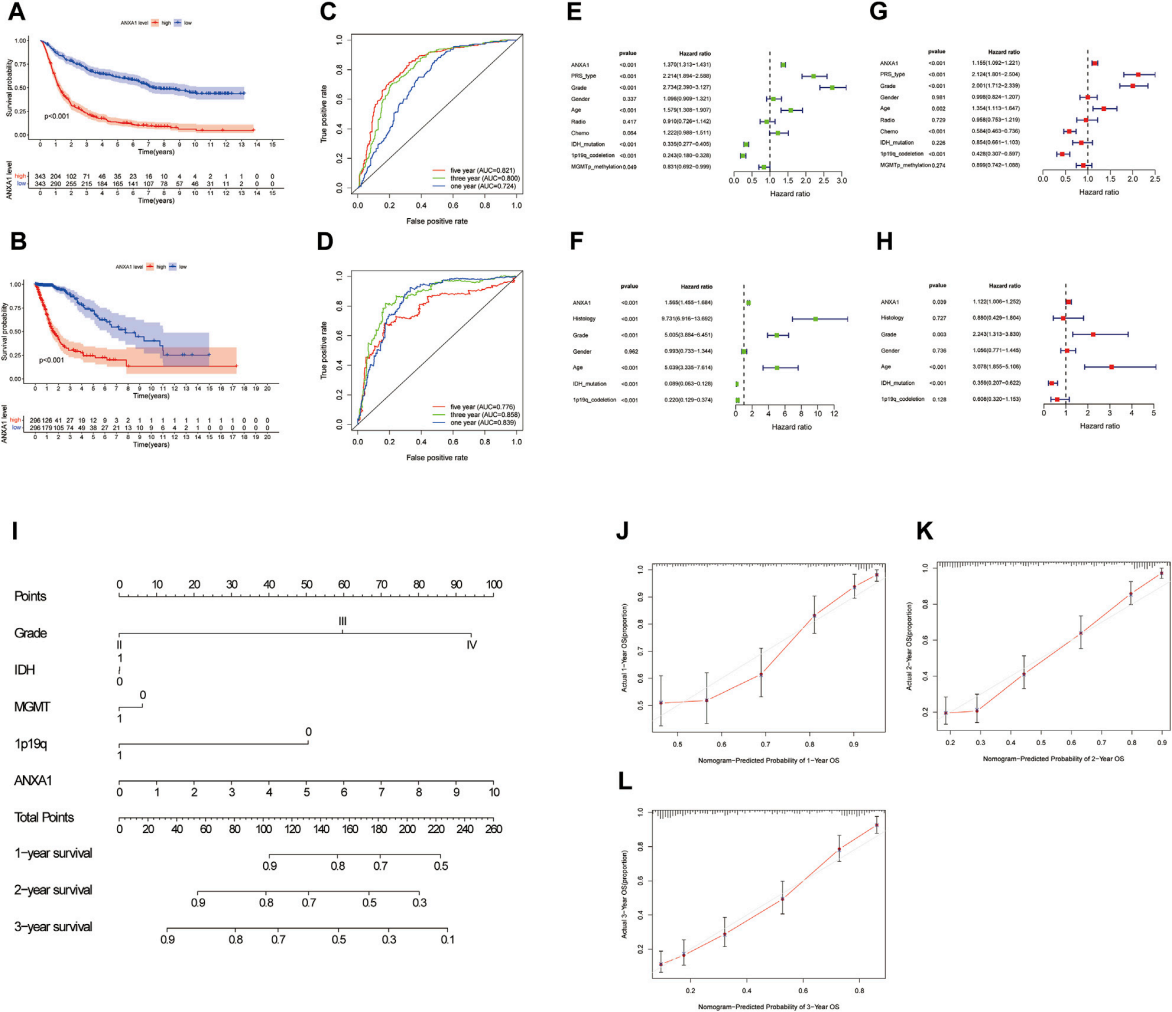
Survival analysis and independent prognostic analysis of the ANXA1 gene in glioma. **(A,B)** Survival analysis of ANXA1. **(C,D)** Survival ROC curve of ANXA1 at 1, 3, and 5 years. **(E,F)** Univariate analysis of ANXA1. **(G,H)** Multivariate analysis of ANXA1. **(I)** Based on the clinical information of the CGGA database, a prognostic nomogram model was constructed. **(J–L)** Calibration curve was constructed according to the CGGA database information. The scores of ANXA1, grade, IDH, MGMT, and 1p19q factors were used to predict the calibration curve of the 1-year, 2-year, and 3-year prognostic nomogram.

The Kaplan–Meier survival method was performed to analyze the survival prognosis using the sample information from the CGGA database, which showed that high expression of ANXA1 had worse prognosis (*p* < 0.001, Figure 3A), and these results were verified by analysis of the TCGA database (*p* < 0.001, Figure 3B). Using the CGGA and TCGA databases, the ROC curve was generated. The ROC curve of ANXA1 for 1-, 3-, and 5-year outcomes had AUC values of 0.724, 0.800, and 0.821, respectively, in the CGGA database (Figure 3C). The ROC curve of the ANXA1 gene was verified in the TCGA database, with AUC values of 0.839, 0.858, and 0.776 for 1-, 3-, and 5-year outcomes, respectively (Figure 3D). Univariate Cox regression analyses showed that the expression of ANXA1 [HR = 1.370; 95% CI (1.313–1.431); *p* < 0.001], type, grade, age, IDH mutation, and 1p19q expression status were significantly correlated with survival prognosis (Figure 3E). Moreover, multivariate Cox regression analyses showed that the expression of ANXA1 [HR = 1.155; 95% CI (1.092–1.221); *p* < 0.001], type, grade, age, chemotherapy, and 1p19q expression status were also correlated with survival prognosis (Figure 3F). The univariate and multivariate regression analyses were verified in the TCGA database (Figures 3G,H). Therefore, these findings indicated that ANXA1 is an independent prognostic indicator of glioma. In addition, based on the clinical characteristics of the CGGA, including grade, IDH mutation, methylation level, 1p19q expression, and ANXA1 expression level, we constructed a nomogram for quantitative prediction (Figure 3I). Finally, we found that the actual and predicted survival times were better matched by the calibration curve in patients with 3-year survival (Figures 3J–L).

### Correlation Between ANXA1 Gene Expression and Clinicopathological Features

**FIGURE 4:**
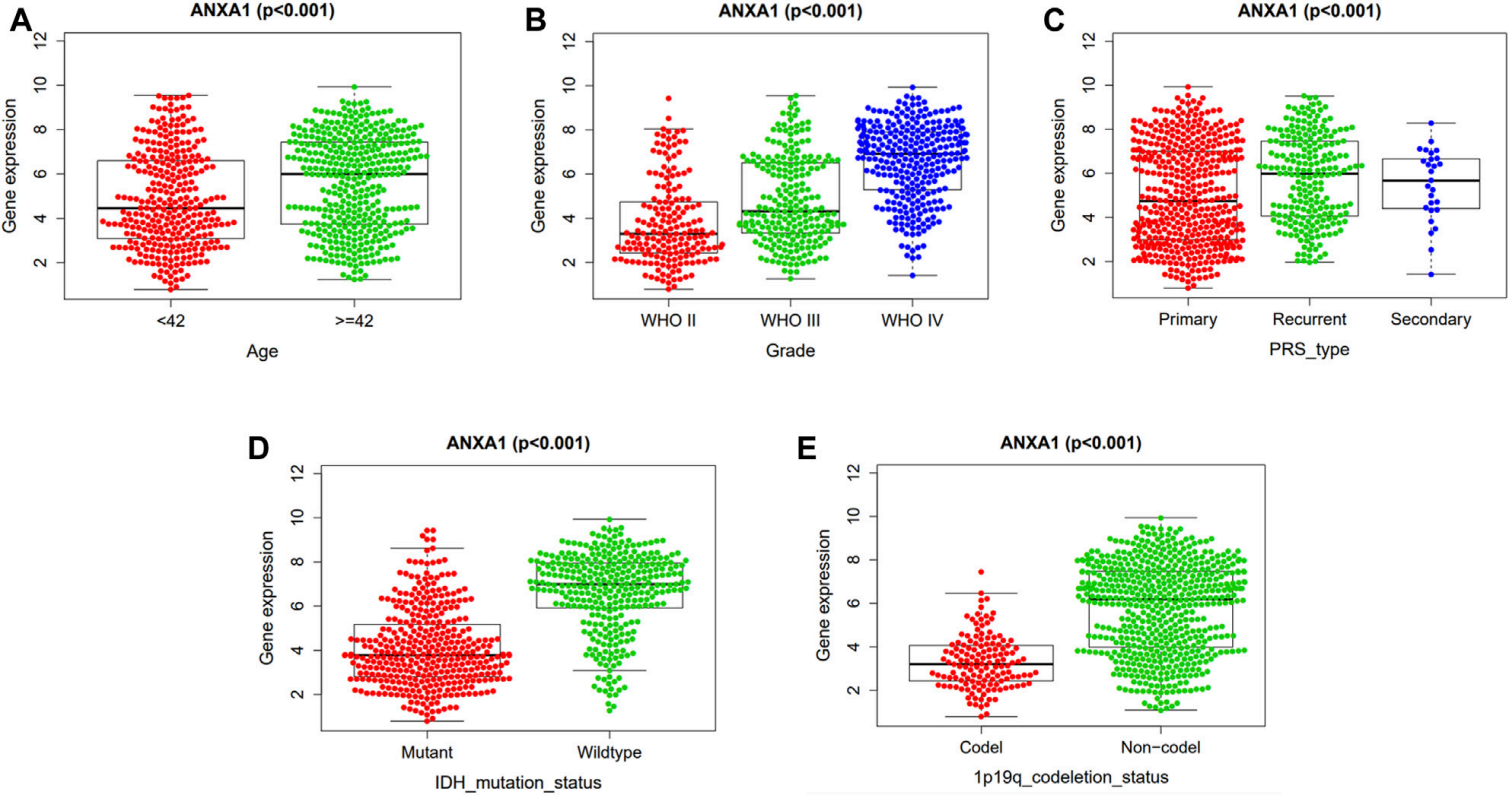
Independent analysis of the ANXA1 gene and clinicopathological features. Based on 686 tumor samples in the CGGA database, the ANXA1 and clinical characteristics were analyzed independently: **(A)** Expression of ANXA1 in age (≥42 years, <42 years). **(B)** Expression of ANXA1 in glioma grade (WHO II, WHO III, and WHO IV). **(C)** Expression of ANXA1 in glioma types (primary, recurrent, and secondary). **(D)** Expression of ANXA1 in IDH mutation state (mutant and wild-type). **(E)** Expression of ANXA1 in 1p19q codeletion status (Codel and non-codel).

The correlation analysis of ANXA1 expression and clinicopathological features demonstrated that ANXA1 was overexpressed in patients older than 42 years (*p* < 0.001, Figure 4A). In addition, the expression of ANXA1 also increased with the increase of tumor grade (*p* < 0.001, Figure 4B); among the type of pathological features, ANXA1 was more highly expressed in recurrent patients (*p* < 0.001, Figure 4C), and ANXA1 expression was low in patients with IDH mutation and combined 1p19q expression (*p* < 0.001, Figures 4D,E).

### Differentially Expressed Genes and Enrichment Analysis of the ANXA1 Gene

**FIGURE 5:**
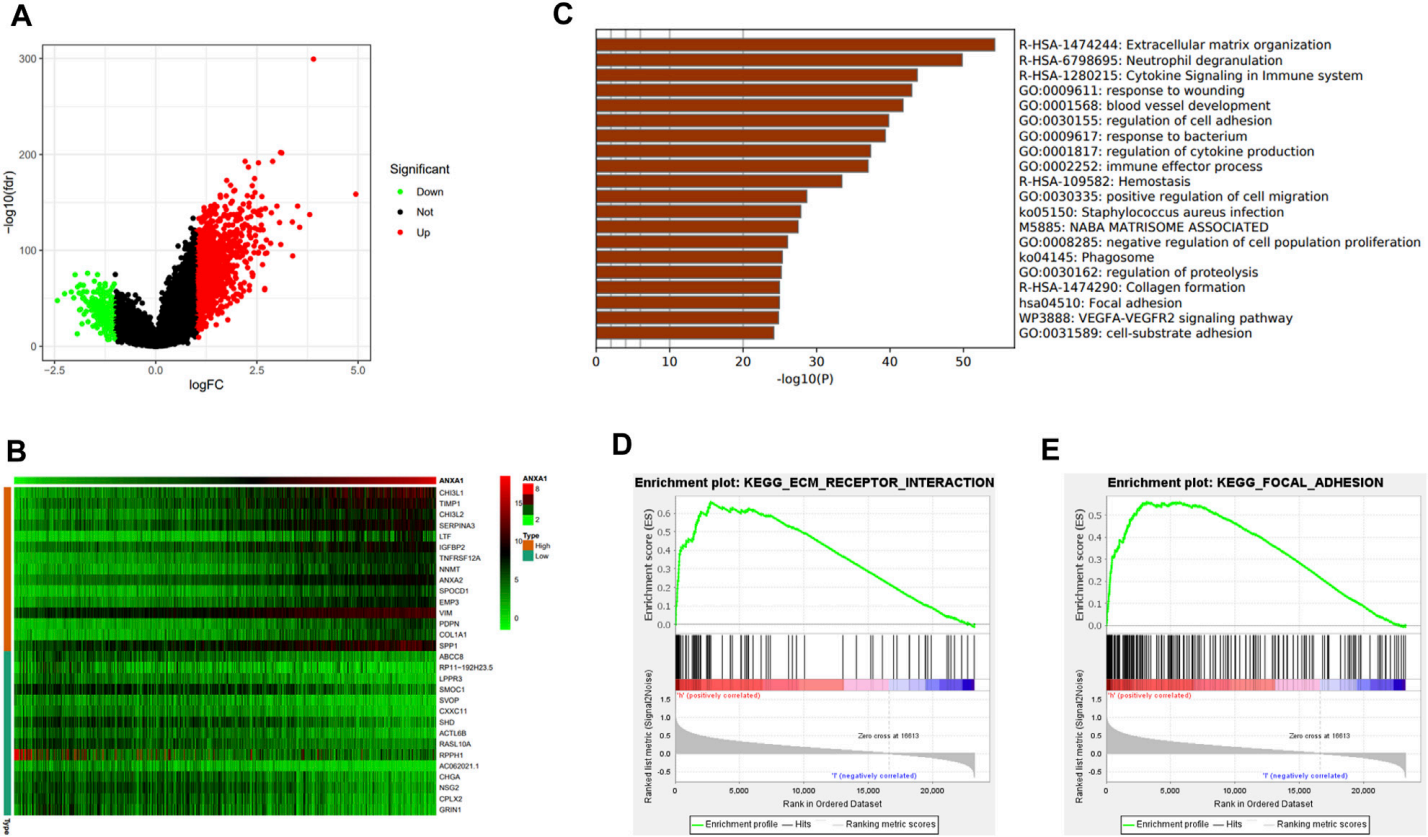
DEG analysis and ANXA1 gene mechanism analysis. **(A)** Visual volcano plot of differentially expressed genes. **(B)** Heat map of DEGs was drawn, showing DEGs that were significantly upregulated and significantly downregulated. **(C)** Visual heat map for the mechanism analysis of DEGs on the Metascape online website. **(D,E)** Enriched ECM–receptor interaction and focal adhesion signal pathway by GSEA.

In the differential expression analysis of ANXA1, 1,256 upregulated genes and 227 downregulated genes were obtained and visualized by a volcano map (Figure 5A), and 30 genes with significant upregulation and downregulation were utilized to generate a heat map (Figure 5B). GSEA indicated that the ANXA1 gene was enriched in the ECM receiver interaction (NSE = 1.96, NOM *p*-val = 0.002, and FDR *q*-val = 0.023) and focal adhesion (NES = 1.99, NOM *p*-val = 0.004, and FDR *q*-val = 0.039) pathways (Figures 5C, D), and Metascape online analysis showed that the DEGs were enriched in the process of ECM and focal adhesion (Figure 5E).

### Construction of Protein–Protein Interaction of Differentially Expressed Genes, Filtration of Core Genes, Core Gene Correlation Analysis, and Prognostic Analysis

**FIGURE 6:**
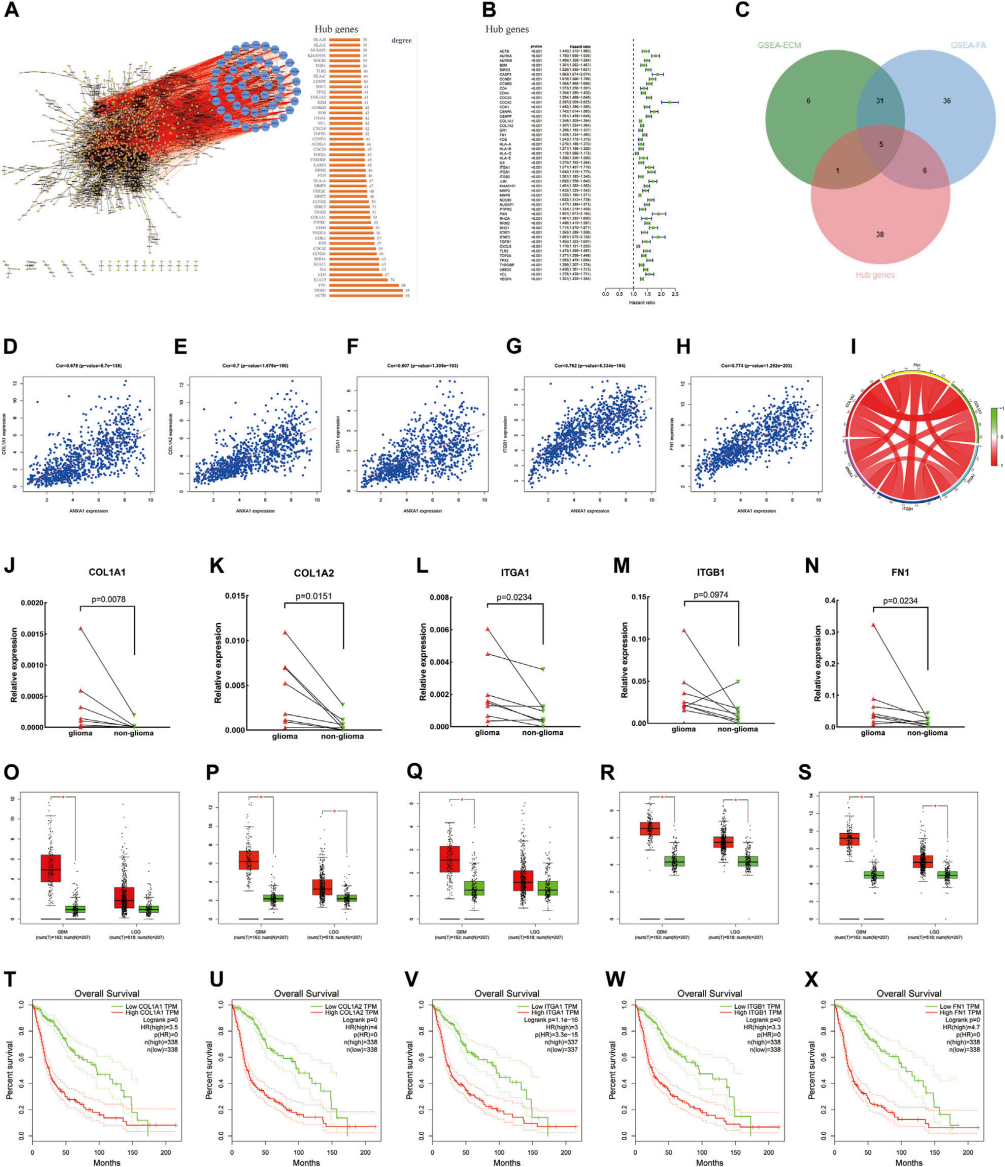
Screening of core genes, gene correlation analysis, and prognostic analysis. **(A)** PPI was constructed by the STRING online website and Cytoscape visualization software, and the top 50 Hub proteins with degree values were screened. **(B)** Univariate regression analysis of the top 50 Hub genes. **(C)** Intersection between ECM–receptor interaction and focal adhesion pathway genes and 50 Hub genes and displayed by the Venn diagram. **(D–I)** Gene correlation circle diagram and scatter diagram. **(J–N)** Relative expression levels of the five core genes were detected by qPCR. **(O–S)** Expression level of core genes in glioma and normal tissues according to the analysis of the GEPIA online website. **(T–X)** High expression level of the core genes has poor prognosis in gliomas.

Based on the STRING online analysis, 1,034 proteins were screened and input into Cytoscape visualization software. The top 50 hub genes were obtained by a degree topology analysis using the cytoHubba plugin (Figure 6A). Univariate regression analysis was performed on the top 50 hub genes, which showed that these genes were risk factors for glioma (*p* < 0.001, Figure 6B). A Venn diagram was used to visualize the intersection of the top 50 hub genes with core genes enriched in the pathway, and five core genes were obtained, namely, COL1A1, COL1A2, FN1, ITGA1, and ITGB1 (Figure 6C). In addition, gene correlation analysis showed that ANXA1 was significantly correlated with COL1A1, COL1A2, FN1, ITGA1, and ITGB1 as indicated by the gene correlation circle diagram and scatter diagram (Figures 6D–I). In the remaining eight glioma samples (included four GBM and four LGG) and their corresponding paracancerous tissues, the relative expression levels of the five core genes were verified by qPCR (Figures 6J–N). The relative expression level is described in the Method section. The statistics were carried out by the paired-sample *t*-test, and the *p* values were 0.0078, 0.0151, 0.0234, 0.0974, and 0.0234. The expression of these genes was higher in gliomas by GEPIA (Figures 6O–S). The higher expression of these genes in gliomas was often accompanied by worse survival prognosis (Figures 6T–X).

## Discussion

Glioma is the most common brain malignant tumor, and its incidence rate has increased in recent years (Wen and Kesari, 2008). Radiotherapy and chemotherapy have become the standard of care in the treatment of gliomas (Nabors et al., 2017). Due to different molecular types and grades of gliomas, GBM, in particular, is often accompanied by high invasiveness and high recurrence. Even after routine surgery and concurrent chemoradiotherapy, the prognosis of glioma patients is still poor (Wang and Jiang, 2013). In recent years, targeted therapy and immunotherapy have improved the prognosis in other common tumors. Although some glioma patients have responded to targeted therapies or immunotherapies, there is still a lack of obvious effect in most patients with glioma (Chen et al., 2017; Xu et al., 2020). Therefore, new predictive targets and potential therapeutic targets for glioma need to be further explored and studied.

In our study, we analyzed the expression of ANXA1 in glioma through the GEO database, which demonstrated that ANXA1 was highly expressed in glioma patients. The GEPIA and Human Protein Atlas online analyses verified these results. Moreover, the overexpression of ANXA1 in glioma was verified by Western blotting analysis. Survival prognosis, ROC curve, and univariate and multivariate Cox regression analyses were performed using the CGGA and TCGA databases, which further showed that ANXA1 played an important role in the development of glioma and supported that ANXA1 was an independent prognostic index of glioma. In addition, the expression of ANXA1 was investigated according to the clinical case characteristics of glioma, and a clinical correlation nomogram was constructed using the CGGA database, which supported that ANXA1 was an important predictor of glioma. Finally, calibration curves were used to verify the matching of actual and predicted survival, and the matching degree of the 3-year survival is higher. Therefore, these findings suggested that ANXA1 warrants further study in glioma.

Previous studies have linked ANXA1 with thrombosis and inflammation (Parente and Solito, 2004; Senchenkova et al., 2019). In addition, it has been reported that ANXA1 is closely related to tumor development and invasion (Boudhraa et al., 2016; Foo et al., 2019). For example, the prognosis of lung cancer patients with a high ANXA1 expression is poor, and the growth of lung cancer cells is reduced by downregulating ANXA1 using small interference RNA (siRNA) (Chuang et al., 2021). Overexpression of ANXA1 promotes metastasis in breast cancer patients, resulting in poor prognosis (de Graauw et al., 2010). Therefore, ANXA1 plays an important role in tumors.

The tumor microenvironment is the internal and external environment for tumor survival. The components of the tumor microenvironment include not only tumor cells but also peripheral blood vessels, ECM, and some molecular signal factors (Hui and Chen, 2015). In the tumor microenvironment, tumor cells change and maintain their own development needs through autocrine and paracrine factors. There is a two-way driving effect between the tumor microenvironment and tumor cell progression, invasion, and metastasis (Meurette and Mehlen, 2018), in which the molecular mechanism is complex. Moreover, due to the complex process of the occurrence, development, invasion, and infiltration of tumor cells (Parker et al., 2020) and the resourcefulness of tumor cells themselves (Dunn et al., 2002), it is difficult to effectively control tumors. GBM, a malignant brain tumor with rapid progression and recurrence, is more difficult to clinically control (Weller et al., 2015). After an analysis of the potential molecular targets of glioma, we found that ANXA1 may play an important role in glioma through ECM–receptor interaction and focal adhesion signal pathways. As an important part of the tumor microenvironment, the ECM has a critical role in tumor occurrence, development, and invasion (Mohan et al., 2020). Focal adhesion is a subcellular structure, which not only has strong adhesion to the ECM but also promotes intracellular reorganization, resulting in a series of dynamic changes in cell function and morphology. Focal adhesion also plays an important role in the process of tumorigenesis, development, and infiltration (Legate et al., 2009). Multifunctional and multiprotein focal adhesion complexes play a key role in the underlying mechanism, which not only promote contact with the ECM but also promote the close conjunction between the ECM and actin cytoskeleton. Therefore, these complexes control the external morphology and internal signals of cells in terms of structure and function to promote cell growth and development, proliferation, differentiation, and motility (Legate and Fässler, 2009; Eke and Cordes, 2015). The main component of the ECM is collagen (Nissen et al., 2019). Type I collagen exists in most connective and embryonic tissues. In general, type I collagen consists of two chains of the alpha 1 chain (COL1A1) and one chain of the alpha 2 chain (COL1A2) (Gelse et al., 2003; Exposito et al., 2010). The alpha 1 chain of type I collagen is encoded by the COL1A1 gene and has been reported to be expressed in a variety of cancers, such as gastric cancer (Wang and Yu, 2018) and glioma (Balbous et al., 2014). COL1A1 is considered to be a marker of mesenchymal osteoblasts (Mori-Akiyama et al., 2003) and is also defined as a glioma endothelial marker selectively expressed in microvessels (Liu et al., 2010). COL1A2 is one of the most abundant collagen types in the human body, and it is involved in the process of angiogenesis. It has been reported that COL1A2 is upregulated in cancer (Greco et al., 2010) and that it promotes the proliferation and invasion of various cancers, such as gastric cancer and pancreatic cancer (Wu et al., 2019; Pan et al., 2021). FN1 is a type of adhesion glycoprotein involved in the ECM function of tumor cells, including cell adhesion, proliferation, and migration (Ko et al., 2020). Integrins play an important role in tumorigenesis, progression, and metastasis because they mediate the adhesion, migration, proliferation, invasion, and tumorigenicity of cancer cells (Pinon and Wehrle-Haller, 2011; Boudjadi et al., 2017). Among them, integrin α1 (ITGA1) and integrin β1 (ITGB1) are important members of the integrin family (Humphries et al., 2006; Hu et al., 2017).

Finally, through the intersection of the enriched core genes of the two pathways and the hub genes, we found that COL1A1, COL1A2, FN1, ITGA1, and ITGB1 were highly correlated with the ANXA1 gene, and these genes were overexpressed in glioma compared to normal brain tissues, which was verified in small samples of gliomas by the qPCR experiment. Through GEPIA, we found that the overexpression of these five core genes had poor prognosis in gliomas. Overall, the present study indicated that these five core genes and the ANXA1 gene participated in the occurrence and development of gliomas and suggested that they may play a synergistic role in the prognosis of gliomas. Therefore, the impact of these genes on gliomas should be further studied to seek the outcomes for glioma patients.

## Conclusion

Glioma is a common primary tumor in the brain. The high recurrence and invasion of gliomas endanger the survival of patients and pose a serious challenge to human health. Therefore, we investigated the expression of the ANXA1 gene in glioma and found that ANXA1 was overexpressed in gliomas. Moreover, we found that the prognosis of glioma patients with a high ANXA1 expression was worse and that ANXA1 was an independent prognostic index of gliomas. The present findings also indicated that ANXA1 may play an important role in the occurrence, development, invasion, and infiltration by affecting the ECM–receptor interaction and the focal adhesion signal pathways. Finally, by using gene correlation analysis, we found that the COL1A1, COL1A2, FN1, ITGA1, ITGB1, and ANXA1 genes may play a synergistic role in glioma patients. Therefore, the present study using public data recommends that ANXA1 may be an important molecular target for glioma. Blocking the overexpression of the ANXA1 gene is likely to improve the prognosis of glioma patients. Because the present study was based on online databases and glioma tissue samples, there were several limitations, indicating that additional clinical experimental studies are needed to improve the reliability of ANXA1 in glioma research.

## Data Availability

The original contributions presented in the study are included in the article/Supplementary Materials, further inquiries can be directed to the corresponding author.
