# Supplementary material for: ANXA1: An Important Independent Prognostic Factor and Molecular Target in Glioma
Source: Front Genet. 2022 May 31;13:851505. doi: 10.3389/fgene.2022.851505 (PMC9193966; doi:10.3389/fgene.2022.851505)
Supplement: Supplementary file 1 [file Table2.DOCX]

Supplementary Material

# Supplementary Tables

**Supplementary Table 1. qPCR Primers**

| **Primer** | **Forward (5’-3’)** | **Reverse (5’-3’)** |
| --- | --- | --- |
| COL1A1 | GCCAAGACGAAGACATCCCA | GCACCATCATTTCCACGAGC |
| COL1A2 | GTGGCAGTGATGGAAGTGTG | AGGACCAGCGTTACCAACAG |
| ITGA1 | CCGAAGAGGTACTTGTTGCAGC | GGCTTCCGTGAATGCCTCCTTT |
| ITGB1 | GCGTGCAGGTGCAATGAAGG | ACAAACACACTGTCCGCAGACG |
| FN1 | TGAAAGACCAGCAGAGGCATAAG | CTCATCTCCAACGGCATAATGG |
| GAPDH | CATGAGAAGTATGACAACAGCCT | AGTCCTTCCACGATACCAAAGT |

**Supplementary Table 2. The clinical information of 23 glioma patients, which including patient age, gender, grade, radiotherapy and chemotherapy, IDH mutation.**

|  | Glioma sample information (n=23) | |
| --- | --- | --- |
|  | **Case** | **Proportion** |
| WHO Grade |  |  |
| II | 7 | 30.43% |
| III | 8 | 34.78% |
| IV | 8 | 34.78% |
| Gender |  |  |
| Male | 15 | 65.22% |
| Female | 8 | 34.78% |
| Age |  |  |
| ≥42 | 15 | 55.2% |
| <42 | 8 | 44.8% |
| IDH mutation |  |  |
| Yes | 6 | 26.09% |
| No | 6 | 26.09% |
| NA | 11 | 47.83% |
| radiotherapy |  |  |
| Yes | 13 | 56.52% |
| No | 2 | 8.70% |
| NA | 8 | 34.78% |
| chemotherapy |  |  |
| Yes | 11 | 47.83% |
| No | 4 | 17.39% |
| NA | 8 | 34.78% |
